# Impact of Surgical Techniques on Anastomotic Stricture in Urinary Diversion: A Systematic Review and Meta-analysis

**DOI:** 10.1016/j.euros.2026.05.002

**Published:** 2026-05-20

**Authors:** Ahmed R. Alfarhan, Akihiro Matsukawa, Angelo Cormio, Marcin Miszczyk, Abdulrahman A. Alhazeem, Abdulrahman S. Alqathtani, Alessandro Dematteis, Ichiro Tsuboi, Navid Roessler, Takafumi Yanagisawa, Jun Miki, Piotr Chłosta, Pierre I. Karakiewicz, Takahiro Kimura, Shahrokh F. Shariat

**Affiliations:** a Department of Urology, Comprehensive Cancer Center, Medical University of Vienna, Vienna, Austria; b Division of Anatomy, Medical University of Vienna, Vienna, Austria; c Department of Urology, Prince Saud Bin Jalawi Hospital, Al Ahsa Health Cluster, Al Ahsa, Saudi Arabia; d Department of Urology, The Jikei University School of Medicine, Tokyo, Japan; e Department of Urology, Azienda Ospedaliero-Universitaria Ospedali Riuniti Di Ancona, Università Politecnica Delle Marche, Ancona, Italy; f Collegium Medicum - Faculty of Medicine, WSB University, Dąbrowa Górnicza, Poland; g Department of Urology, King Fahad Hospital Hofuf, Al Ahsa Health Cluster, Al Ahsa, Saudi Arabia; h Department of Urology, Ministry of Health Saudi Arabia, Second Health cluster, Riyadh, Saudi Arabia; i Department of Surgical Sciences, Division of Urology, AOU Citt‘a della Salute e della Scienza at Molinette Hospital and University of Turin, Turin, Italy; j Department of Urology, Shimane University Faculty of Medicine, Shimane, Japan; k Department of Urology, University Medical Center Hamburg-Eppendorf, Hamburg, Germany; l Department of Urology, Jagiellonian University Medical College, Krakow, Poland; m Cancer Prognostics and Health Outcomes Unit, Division of Urology, University of Montréal Health Center, Montréal, Canada; n Department of Urology, Semmelweis University, Budapest, Hungary; o Department of Urology, the University of Texas Southwestern Medical Center, Dallas, TX, USA; p Department of Urology, Weill Cornell Medical College, New York, NY, USA; q Department of Urology, Second Faculty of Medicine, Charles University, Prague, Czechia; r Division of Urology, Hourani Center for Applied Scientific Research, Al-Ahliyya Amman University, Amman, Jordan; s Karl Landsteiner Institute of Urology and Andrology, Vienna, Austria; t Research Center for Evidence Medicine, Urology Department Tabriz University of Medical Sciences, Tabriz, Iran

**Keywords:** Ureter-enteric strictures, Radical cystectomy, Urinary diversion, Robot-assisted radical cystectomy, Open radical cystectomy, Wallace technique, Bricker technique, Surgical techniques, Urological complications, Anastomotic strictures

## Abstract

**Context:**

Benign ureteral-ileal anastomotic stricture (UIAS) is a common complication of urinary diversion (UD). Many surgical techniques have been tested to mitigate its risk; however, an optimal strategy is yet to be established. **Objective:** In this study, we evaluated the impact of various surgical techniques on UIASs in UD.

**Evidence acquisition:**

In July 2024, we searched MEDLINE, Embase, and Web of Science. Random-effects pairwise meta-analyses of risk ratios (RRs) were performed to compare the risk of UIAS between various surgical techniques. The risk of bias was assessed using RoB2, ROBINS-I tools, and Newcastle-Ottawa Scale. (PROSPERO: CRD42024574087).

**Evidence synthesis:**

A total of 46 studies (*N* = 11 732 patients) were included in meta-analysis. Robotic surgery was significantly associated with a higher risk of UIAS compared with open surgery (RR: 1.47; 95% confidence interval [CI]: 1.24–1.76; *p* < 0.001). Intracorporeal UD (ICUD) was significantly associated with an increased risk of UIAS compared with extracorporeal UD (ECUD; RR: 1.34; 95% CI: 1.05–1.72; *p* = 0.020); however, in an analysis limited to robotic surgery, no significant difference was observed between ICUD and ECUD (RR: 1.06; 95% CI: 0.73–1.54; *p* = 0.7). No significant differences were observed using Wallace versus Bricker technique (RR: 0.68; 95% CI: 0.40–1.18; *p* = 0.17), Indocyanine green dye (RR: 0.44; 95% CI: 0.12–1.56; *p* = 0.2), stent-free approach (RR: 0.60; 95% CI: 0.30–1.21; *p* = 0.15), or single versus double J stents (RR: 0.93; 95% CI: 0.41–2.10; *p* = 0.9).

**Conclusion:**

The risk of UIAS is significantly higher in the robotic surgery approach, which should prompt more cautious follow-up. Moreover, it was significantly higher in patients with ECUD, while the commonly used UD methods were not significantly associated with the risk of UIAS. Further prospective studies are needed to validate these findings.

## Take Home Message

Robotic-assisted radical cystectomy is linked with a 47% higher risk of ureteral-ileal anastomotic strictures compared to open surgery, highlighting the need for vigilant postoperative monitoring. Intracorporeal urinary diversion shows a modestly increased stricture risk versus extracorporeal diversion overall, but this difference disappears when limited to robotic cases, suggesting selection and surgical approach interplay. No clear benefit emerged for Wallace versus Bricker anastomotic techniques in reducing strictures, although a consistent trend favors Wallace—underscoring the importance of surgeon familiarity and technique refinement. Intraoperative indocyanine green perfusion imaging demonstrated a non-significant trend toward fewer strictures, supporting its role in assessing ureteral blood flow despite limited direct impact on stricture rates. Whether stents are used (ie, single J, double J, or none), stricture rates remain comparable, indicating that stent choice should be driven by surgeon preference and patient factors rather than stricture prevention alone. Refluxing versus non-refluxing ureteral implantation yielded conflicting outcomes across small series; larger, standardized trials are needed before altering current anastomotic policies. Running versus interrupted suture techniques showed conflicting evidence on stricture risk, pointing to the critical role of meticulous surgical handling over suture pattern alone. Future research should prioritize prospective, randomized studies that control surgeon experience, patient characteristics, and standardized anastomotic protocols to definitively identify strategies that minimize ureteroenteric strictures.

ADVANCING PRACTICE **What does this study add?**

This comprehensive systematic review and meta-analysis provide the most up-to-date evaluation of the impact of various surgical techniques on anastomotic strictures in urinary diversion (UD), addressing a critical issue in urological practice. The findings challenge conventional assumptions by demonstrating significant differences in stricture risk between robot-assisted and open radical cystectomy as well as between intracorporeal and extracorporeal UD, offering actionable insights for surgical decision-making. By synthesizing data from many studies, this research provides evidence-based guidance that can directly influence clinical practice, leading to improved surgical strategies, reduced postoperative complications, and enhanced long-term outcomes for patients undergoing UD.

**Clinical Relevance**

UIAS affects approximately 10% of patients following radical cystectomy and can lead to obstruction, pyelonephritis, and renal impairment requiring reintervention. Robot-assisted surgery was associated with a 47% higher risk of UIAS compared with open surgery (RR 1.47; 95% CI 1.24–1.76), and intracorporeal diversion with a 34% higher risk overall, though this difference disappeared when the analysis was restricted to robotic cases. These findings should prompt more cautious postoperative surveillance in patients undergoing robotic urinary diversion, and encourage efforts to refine anastomotic technique in this setting. The lack of significant benefit from commonly used modifications underscores the need for prospective, randomized trials. Associate Editor: Véronique Phé.

**Patient Summary**

We studied different surgical techniques used in urinary diversion (UD) to understand their impact on complications, particularly on anastomotic strictures. Our findings suggest that some surgical approaches may increase the risk of strictures, which can lead to difficulties with urinary flow and require further medical intervention. By identifying safer techniques, surgeons can reduce complications, improve recovery, and ensure better long-term outcomes for patients undergoing UD.

## 1 Context

Urinary diversion (UD) following radical cystectomy (RC) is among the most complex surgical procedures in urology. Despite advancements and refinements over the years, most patients experience at least one complication, with 20% requiring further intervention after RC [1], [2]. Benign ureteral-ileal anastomotic stricture (UIAS) is one of the most common complications, that affects approximately 10% of patients [3], [4]. While most patients with UIAS remain asymptomatic, the condition can progress to symptomatic obstruction, urinary stones, complex pyelonephritis, or renal impairment, which may require further interventions. Therefore, preventing UIAS during surgery is crucial. To reduce this complication, several approaches have been investigated, including robotic-assisted RC (RARC) versus open RC (ORC), intracorporeal versus extracorporeal approach to UD (ICUD vs ECUD), ureteroenteric anastomosis method (Bricker technique and Wallace technique), the use of various stents (ie, Single J [SJ] and Double J [DJ] stents), and indocyanine green (ICG) for perfusion assessment; however, the results still remain controversial [5], [6], [7], [8], [9], [10].

Although several meta-analyses have been conducted on individual topics, none have comprehensively reviewed various surgical techniques in a single study. **Objective:** In this study, we conducted a systematic review and meta-analysis to evaluate the impact of various surgical techniques on UIAS in UD.

## 2 Methodology

The protocol for this study has been registered with the International Prospective Register of Systematic Reviews database (PROSPERO: CRD42024574087). We used the Preferred Reporting Items for Systematic Reviews and Meta-Analyses (PRISMA) and A MeaSurement Tool to Assess systematic Reviews (AMSTAR) checklists to ensure transparent reporting of our meta-analysis (Fig. S1) [11], [12].

### 2.1 Study selection and characteristics

**Fig. 1:**
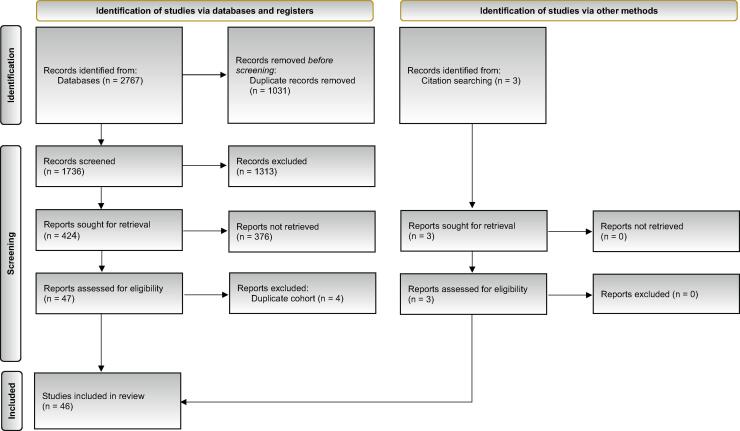
PRISMA 2020 flow diagram for new systematic reviews which included searches of databases, registers, and other sources.

On July 28, 2024 we searched MEDLINE (via PubMed), Embase, and Web of Science Core Collection databases to identify studies assessing the incidence of strictures following ureteral-intestinal anastomosis after RC. Detailed search strategy is presented in the Supplementary Material. Two authors independently screened reports for eligibility at both the abstract and full-text stages. Any disagreements raised were resolved through consensus with coauthors (see Fig. 1).

### 2.2 Inclusion/Exclusion criteria

The PICOS (Population, Intervention, Comparator, Outcome and Study Design) framework was used to formulate the research question [13]. The patients who underwent UD following RC (Population) with various types of surgical techniques (Interventions) compared to other types of surgical techniques (Comparisons) were included in this study. The primary endpoint was the incidence of UIAS (Outcomes). Our exclusion criteria were reviews, editorial comments, case reports, replies to authors, meeting abstracts, non-human studies, and articles not published in English. Additionally, studies that included patients with anastomotic strictures caused by malignancies, metachronous cancer recurrence, or other cancer-related conditions were also excluded. Two authors independently extracted data from the studies, including basic study data and outcome measures (ie, risk ratios [RRs] for UIAS between different surgical methods). Any disagreements raised were resolved through consensus with coauthors.

### 2.3 Risk of Bias

We evaluated the quality of the studies and assessed the risk of bias using the Risk of Bias in Non-randomized Studies–of Interventions tool (ROBINS-I) for non-randomized studies, and the Risk of Bias 2 tool for randomized trials (RoB2), as outlined in the Cochrane Handbook for systematic reviews of interventions. Similarly, the Newcastle-Ottawa Scale (NOS) was used to assess the case-controlled studies. These assessments were conducted independently by two authors [14], [15], [16].

### 2.4 Statistical analysis

Standard pairwise meta-analyses were performed using the “meta” package in R Software version 4.4.2 (R Foundation for Statistical Computing, Vienna, Austria), following Cochrane Collaboration guidelines. We applied a random-effects model using the DerSimonian–Laird method to estimate the between-study variance (τ^2^) and employed inverse-variance weighting to pool the study-specific effect sizes. We selected the DerSimonian–Laird approach as it is widely accepted and provides a transparent method-of-moments estimator for heterogeneity, yielding conservative overall estimates when variability exists across studies [17]. Pooled RRs with corresponding 95% confidence intervals (CIs) were calculated using the total number of patients and number of events, and the results were presented using forest plots. Subgroup analyses were conducted based on the study design (comparing methods). To minimize heterogeneity, we additionally conducted an exploratory subgroup analysis comparing ICUD and ECUD restricted to RARC. Meta-analyses were only performed when at least two studies were available for comparison. Heterogeneity among the included studies was assessed using Cochran’s Q test. Publication bias was evaluated using funnel plots, and Egger’s test was applied when more than 10 studies were available for each analysis. Funnel plots were generated for all comparisons to ensure transparency. However, as recommended by current systematic review guidelines, interpretation of funnel plot symmetry was limited to comparisons including ≥10 studies, because asymmetry is difficult to assess when fewer studies are available. The statistical significance threshold was set at *p* < 0.05, and all tests were two-sided. When a study recorded no events in one treatment arm, we added 0.5 to each zero count to calculate the risk estimates properly. When significant heterogeneity was present (*p* < 0.05), subgroup analyses by study design and surgical approach (ICUD vs ECUD) were performed. Studies reporting outcomes on a per-ureter basis rather than per-patient were excluded from meta-analysis to ensure consistency in the measurement and pooling of clinical endpoints.

## 3 Evidence acquisition

### 3.1 Study selection and characteristics

Out of 1736 individual records screened, 46 studies (*N* = 11 732 patients) were found to be eligible for this study, including four RCTs (*n* = 325) [18], [19], [20], [21], four prospective cohort studies (*n* = 375) [22], [23], [24], [25], 34 retrospective cohort studies (*n* = 7729) [3], [4], [7], [9], [26], [27], [28], [29], [30], [31], [32], [33], [34], [35], [36], [37], [38], [39], [40], [41], [42], [43], [44], [45], [46], [47], [48], [49], [50], [51], [52], [53], [54], [55], one retrospective population-based study (*n* = 1781), and three retrospective case-control studies (*n* = 1522) [56], [57], [58], [59]. The median age of participants across the included studies ranged from the early 50s to 80s. The study periods spanned from 1985 to 2023. Most studies originated from Germany (five studies; *n* = 1564) and the United States (17 studies; *n* = 6350). Three studies each were conducted in China (*n* = 381) and Egypt (*n* = 222), whereas two studies each were reported from France (*n* = 249), Italy (*n* = 179), Japan (*n* = 517), the United Kingdom (*n* = 295), and Turkey (*n* = 171). Additional studies were also identified from Denmark, Greece, India, the Netherlands, Pakistan, Serbia, Spain, and Switzerland. Basic study characteristics are summarized in Table 1.

**Table 1:** Systematic review of different comparisons for the risk of ureteral-ileal anastomotic stricture

Author	Country	Year	Study Design	Period	Summary of Numbers	Age, Yr	Male sex (%)	Follow-up (mo)	UIA
RARC vs ORC									
Anderson et al. [4]	USA	2013	Retrospective Cohort Study	2007–2011	Total: 478 RARC: 103 ORC: 375	RARC: 69 (IQR: 60–74) ORC: 68 (IQR: 61–74)	RARC: 98:95% ORC: 288:77%	8 (IQR: 4–17)	Total: 45 RARC: 13 ORC: 32
Huang et al. [18]	USA	2022	RCT	2010–2013	Total: 118 RARC: 60 ORC: 58	RARC: 66 (IQR: 60–71) ORC: 65 (IQR: 58–69)	RARC: 51:85% ORC: 42 :72%	61 (IQR: 29–79)	Total: 5 RARC: 0 ORC: 5
Brandt et al. [57]	Denmark	2024	Retrospective Case-control Study	2012–2018	Total: 693 RARC: 249 ORC: 444	72 (IQR: 66–78)	505:73%	24 (IQR: 12–31)	Total: 109 RARC: 51 ORC: 58
Beirnaert et al. [24]	France	2022	Prospective Cohort Study	2017–2021	Total: 123 RARC: 38 ORC: 85	RARC: 54±15 ORC: 56±14	RARC: 33:87% ORC: 53:62%	17 (IQR: 5–41)	Total: 23 RARC: 3 ORC: 20
Haudeber et al. [29]	France	2022	Retrospective Cohort Study	2004–2020	Total: 126 RARC: 69 LRC: 26 ORC: 31	52±14	55:44%	23 (IQR: 2–281)	Total: 9 RARC: 6 LRC: 1 ORC: 2
Das et al. [58]	USA	2024	Retrospective Case-control Study	2015–2022	Total: 366 RARC: 206 ORC: 160	69 (IQR: 61–76)	NA	14 (IQR: 6–33)	Total: 35 RARC: 18 ORC: 17
Goh et al. [56]	USA	2020	Population-based Retrospective Study	2009–2014	Total: 1781 RARC: 332 ORC: 1449	RARC 66–70: 31% 71–75: 31% 76–80: 22% >80:16% ORC 66–70: 31% 71–75: 29% 76–80: 22% >80: 18%	RARC: 279:84% ORC: 1158:80%	NA	Total: 256 RARC: 72 ORC: 184
Reesink et al. [32]	Netherland	2021	Retrospective Cohort Study	2012–2017	Total: 279 RARC: 87 ORC: 192	RARC: 66 (IQR: 58–73) ORC: 68 (IQR: 62–75)	RARC: 58:67% ORC: 138:72%	50 (IQR:NA)	Total: 47 RARC: 22 ORC: 25
Faraj et al. [31]	USA	2021	Retrospective Cohort Study		Total: 573 RARC + ICUD: 39 RARC + ECUD: 197 ORC: 337	RARC + ICUD: 76 (IQR: 73–81) RARC + ECUD: 71 (IQR: 66–77) ORC: 71 (IQR: 64–77)	NA	RARC + ICUD: 71 RARC + ECUD: 70 ORC: 55 (IQR: NA)	Total: 47 RARC: 20 ORC: 27
Lone et al. [7]	USA	2021	Retrospective Cohort Study	2010–2018	Total: 644 RARC + ICUD: 191 RARC + ECUD: 260 ORC: 193	67±10	534 (83%)	32 (IQR: 12–56)	Total: 103 RARC: 78 ORC: 25
ICUD vs ECUD									
Tuna et al. [50]	Turky	2023	Retrospective Cohort Study	2014–2021	Total: 30 ICUD: 17 ECUD: 13	ICUD with using ICG: 69 (IQR: 67–78) ICUD without using ICG: 70.5 (IQR: 65–73) ECUD: 59 (IQR: 58–63)	27:90%	NA	Total: 25 RARC + ICUD: 2 RARC + ECUD: 3
Faraj et al. [31]	USA	2021	Retrospective Cohort Study		Total: 573 RARC + ICUD: 39 RARC + ECUD: 197 ORC: 337	RARC + ICUD: 76 (IQR: 73–81) RARC + ECUD: 71 (IQR: 66–77) ORC: 71 (IQR: 64–77)	NA	RARC + ICUD: 71 RARC + ECUD: 70 ORC: 55 (IQR: NA)	Total: 20 RARC + ICUD: 1 RARC + ECUD: 19
Lone et al. [7]	USA	2021	Retrospective Cohort Study	2010–2018	Total: 644 RARC + ICUD: 191 RARC + ECUD: 260 ORC: 193	67±10	534:83%	32 (IQR: 12–56)	Total: 78 RARC + ICUD: 36 RARC + ECUD: 42
Beirnaert et al. [24]	France	2022	Prospective Cohort Study	2017–2021	Total: 123 ICUD: 38 ECUD: 85	RARC: 54±15 ORC: 56±14	RARC: 33:87% ORC: 53:62%	17 (IQR: 5–41)	Total: 23 ICUD: 3 ECUD: 20
Brandt et al. [57]	Denmark	2024	Retrospective Case-control Study	2012–2018	Total: 249 RARC + ICUD: 223 RARC + ECUD: 26	72 (IQR: 66–78)	505:73%	24 (IQR: 12–31)	Total: 51 RARC + ICUD: 46 RARC + ECUD: 5
Kanno et al. [39]	Japan	2020	Retrospective Cohort Study	2003–2019	Total: 144 ICUD: 72 ECUD: 72	ICUD: 72 (IQR: 66–76) ECUD: 73 (IQR: 68–76)	ICUD: 51:71% ECUD: 51:71%	ICUD:28 (IQR: 13–42) ECUD:23 (IQR: 12–41)	Total: 2 ICUD: 0 ECUD: 2
Morizane et al. [28]	Japan	2024	Retrospective Cohort Study	2018–2021	Total: 373 ECUD: 150 ICUD: 155 HUD: 68	ECUD: 72±9 ICUD: 71±8 HUD: 72±8	ECUD: 108:72% ICUD: 125:81% HUD: 56:82%	17 days (IQR: 7.5–23.6 days)	Total: 19.3 RARC + ICUD: 11.9 RARC + ECUD: 7.4
Reesink et al. [32]	Netherland	2021	Retrospective Cohort Study	2012–2017	Total: 279 ICUD: 87 ECUD: 192	RARC: 66 (IQR: 58–73) ORC: 68 (IQR: 62–75)	RARC: 58:67% ORC: 138:72%	50 (IQR: NA)	Total: 47 ICUD: 22 ECUD: 25
Tan et al. [42]	UK	2019	Retrospective Cohort Study	2015–2017	Total: 127 ICUD: 59 ECUD: 68	ICUD: 69 (IQR: 62–74) ECUD: 71 (IQR: 66–76)	ICUD: 49:79% ECUD: 60:86%	ICUD:4 ECUD:14 (IQR: NA)	Total: 7 ICUD: 2 ECUD: 5
Wallece vs Bricker									
Adnan et al. [38]	Pakistan	2022	Retrospective Cohort Study	2009–2018	Total: 116 Wallace: 43 Bricker: 73	54±11	97:84%	48 (IQR: 24–94)	Total: 19 Wallace: 8 Bricker: 11
Al-Nader et al. [27]	Germany	2024	Retrospective Cohort Study	2004–2022	Total: 740 Wallace: 486 Bricker: 254	66±10	609:82%	25 (IQR: NA)	Total: 55 Wallace: 30 Bricker: 25
Can et al. [34]	Turky	2024	Retrospective Cohort Study	2014–2022	Total: 141 Wallace: 60 Bricker: 42 Hybrid: 39	Wallace: 66 (IQR: 32–81) Bricker: 65 (IQR: 40–75) Hybrid: 66 (IQR: 48–77)	Wallace: 52:87% Bricker: 37:88% Hybrid: 32:82%	Wallace:20 (IQR: 10–71) Bricker:18 (IQR: 10–31) Hybrid:18 (IQR: 12–36)	Total: 21 Wallace: 7 Bricker: 11 Hybrid: 3
Christoph et al. [51]	Germany	2019	Retrospective Cohort Study	2009–2014	Total: 140 Wallace: 65 Bricker: 75	Wallace: 71 Bricker: 71 (IQR: NA)	Wallace: 46:70% Bricker: 50:67%	Wallace:17 Bricker:37 (IQR: NA)	Total: 24 Wallace: 5 Bricker: 19
Djordjevic et al. [44]	Serbia	2021	Retrospective Cohort Study	NA	Total: 60 Wallace: 30 Bricker: 30	Wallace: 68±7 Bricker: 63±7	Wallace: 24:80% Bricker: 22:73%	24 (IQR: NA)	Total: 2 Wallace: 0 Bricker: 2
Evangelidis et al. [33]	USA	2006	Retrospective Cohort Study	1997–2003	Total: 198 Wallace: 112 Bricker: 86	Wallace: 62 Bricker: 66 (IQR: NA)	Wallace: 78:70% Bricker: 55:64%	Wallace: 19 Bricker: 21 (IQR: NA)	Total: 8 Wallace: 5 Bricker: 3
Kouba et al. [26]	USA	2007	Retrospective Cohort Study	2001–2005	Total: 184 Wallace: 90 Bricker: 94	Wallace: 67±12 Bricker: 66±12	Wallace: 75% Bricker: 78%	Wallace: 33±21 Bricker: 34±21	Total: 7 Wallace: 0 Bricker: 7
Liu et al. [22]	China	2014	Prospective Cohort Study	2009–2011	Total: 99 Wallace: 46 Bricker: 53	Wallace: 63±9 Bricker: 62±9	Wallace: 38:83% Bricker: 44:83%	Wallace: 26±10 Bricker: 26±10	Total: 6 Wallace: 2 Bricker: 4
ICG VS NON-ICG									
Ahmadi et al. [53]	USA	2019	Retrospective Cohort Study	2014–2017	Total: 179 ICG: 47 Non-ICG: 132	ICG: 68 (IQR: 44–92) Non-ICG: 72 (IQR: 45–89)	ICG: 40:85% Non-ICG: 108:82%	ICG: 12 (IQR: 2–20) Non-ICG: 14 (IQR: 1–42)	Total: 14 ICG: 0 Non-ICG: 14
Doshi et al. [37]	USA	2020	Retrospective Cohort Study	2017–2019	Total: 61 ICG: 31 Non-ICG: 30	71 (IQR: 63–74)	48:79%	ICG: 16 (IQR: 12–18) Non-ICG: 23 (IQR: 7–29)	Total: 6 ICG: 1 Non-ICG: 5
Tuna et al. [50]	Turky	2023	Retrospective Cohort Study	2014–2021	Total: 17 ICG: 7 Non-ICG: 10	ICUD with using ICG: 69 (IQR: 67–78) ICUD without using ICG: 70.5 (IQR: 65–73) ECUD: 59 (IQR: 58–63)	27:90%	NA	Total: 2 ICG: 0 Non-ICG: 2
Carbonell et al. [55]	Spain	2024	Retrospective Cohort Study	2018–2023	Total: 221 ICG: 79 Non-ICG: 142	ICG: 69 (IQR: 63–74) Non-ICG: 69 (IQR: 62–75)	ICG: 70:89% Non-ICG: 119:84%	21 (IQR: 11–35)	Total: 41 ICG: 15 Non-ICG: 26
Shen et al. [40]	USA	2019	Retrospective Cohort Study	2015–2018	Total (Ureters): 186 ICG: 93 Non-ICG: 93	ICG: 69 (IQR: 65–76) Non-ICG: 74 (IQR: 62–80)	ICG: 76:82% Non-ICG: 75:81%	ICG: 12 Non-ICG: 24 (IQR: NA)	Total: 7 ICG: 0 Non-ICG: 7
Stent vs Stent-free									
Mahmood et al. [35]	USA	2024/2023	Retrospective Cohort Study	2005–2023	Total: 488 Stent-free: 122 Stent: 366	Stent-free: 70 (IQR: 65–77) Stent: 71 (IQR: 64–77)	Stent-free: 92:75% Stent: 276:75%	21 (IQR: 12–34)	Total: 72 Stent-free: 9 Stent: 63
Mattei et al. [19]	Switzerland	2008	RCT	NA	Total: 54 Stent-free: 25 Stent: 29	68 (IQR: 45–85)	NA	NA	Total: 3 Stent-free: 0 Stent: 3
Mullins et al. [48]	USA	2012	Retrospective Cohort Study	2003–2007	Total: 192 Stent-free: 123 Stent: 69	66.5 (IQR: 42–85)	159:83%	25 (IQR: 1–156)	Total: 19.1 Stent-free: 9.1 Stent: 10
Regan et al. [43]	USA	1985	Retrospective Cohort Study	NA	Total: 362 Stent-free: 236 Stent: 126	63 (IQR: NA)	260:72%	Stent-free: 17 SJ: 12 Other stent: 13 (IQR: NA)	Total: 10.86 Stent-free: 3 Stent: 13.86
Single J vs Double J									
Ibrahim et al. [9]	UK	2024	Retrospective Cohort Study	2014–2023	Total: 168 SJ: 40 DJ: 128	SJ: 67 (IQR: 49–75) DJ: 67 (IQR: 51–76)	SJ: 30:75% DJ: 103:80%	NA	Total: 5 SJ: 3 DJ: 2
Osman et al. [20]	Egypt	2016	RCT	2008–2012	Total: 93 SJ: 48 DJ: 45	SJ: 56±9 DJ: 56±7	SJ: 43:54% DJ: 36:46%	3 (IQR: NA)	Total: 1 SJ: 1 DJ: 0
Hakim et al. [23]	Egypt	2016	Prospective Cohort Study	2010–2012	Total: 69 SJ: 33 DJ: 36	58 (IQR: NA)	NA	DJ: 28±3 SJ: 29±4	Total: 2 SJ: 2 DJ: 0
Micali et al. [30]	Italy	2001	Retrospective Cohort Study	1994–1998	Total: 77 SJ: 45 DJ: 32	63 (IQR: 49–73)	NA	30 (IQR: NA)	Total: 16 SJ: 9 DJ: 7
Varkarakis et al. [52]	Greece	2005	Retrospective Cohort Study	1994–2002	Total: 43 SJ: 30 DJ: 13	61 (IQR: 51–70)	40:93%	SJ: 23 (IQR: 12–96) DJ: 20 (IQR: 12–30)	Total: 3 SJ: 2 DJ: 1
Reflexing vs Non-reflexing ureter									
Pantuck et al. [49]	USA	2000	Retrospective Cohort Study	1990–1998	Total (Ureters): 116 Non-Reflexing : 60 Reflexing (Nesbit): 56	63 (IQR: 40–85)	NA	36 (IQR: 2–103)	Total (Ureters): 9 Non-Reflexing : 8 Reflexing (Nesbit): 1
Roth et al. [54]	Germany	1997	Retrospective Cohort Study	1991–1995	Total: 120 Reflex Nesbit: 46 Non-Reflex Le Duc: 74	63 (IQR: 35–78)	120:100%	Reflex Nesbit: 14 (IQR: 3–25) Non-Reflex Le Duc: 25 (IQR: 5–72)	Total: 32 Reflexing (Nesbit): 3 Non-Reflexing (Le Duc): 29
Hautman et al. [41]	Germany	2006	Retrospective Cohort Study	1995–2003	Total: 106 Reflexing (Chimney): 37 Non-reflexing (Le Duc): 69	64 (IQR: 36–89)	82:78%	24 (IQR: NA)	Total: 21 Reflexing (Chimney): 11 Non-reflexing (Le Duc): 10
De Carli et al. [47]	Italy	1997	Retrospective Cohort Study	1989–1995	Total: 102 Non-reflex: 50 Nesbit: 52	NA	NA	34 (IQR: 6–77)	Total: 12 Non-reflex: 6 Nesbit: 6
Running vs Interrupted									
Large et al. [45]	USA	2013	Retrospective Cohort Study	2007–2010	Total: 158 Interrupted: 149 Running: 109	Interrupted: 69 (IQR: 67–70) Running: 68 (IQR: 65–70)	Interrupted: 116:78% Running: 89:82%	Interrupted: 17 (IQR: 6–43) Running: 12 (IQR: 4–24)	Total: 52 Interrupted: 25 Running: 27
Retrosigmoid transposition									
Wang et al. [25]	China	2022	Prospective Cohort Study	2018–2020	Total: 84 Anteriorsigmoid: 30 Retrosigmoid: 54	69±9	64:76%	Anteriorsigmoid:29 (IQR: 13–39) Retrosigmoid:28 (IQR: 12–37)	Total: 2 Anteriorsigmoid: 2 Retrosigmoid: 0
ST vs ET									
Wiesner et al. [36]	Germany	2007	Retrospective Cohort Study	1985–2003	Total: 458 ST: 418 ET: 40	ST: 48 ET: 38 (IQR: NA)	ST: 260:62% ET: 22:55%	89 (IQR: NA)	Total: 51 ST: 48 ET: 3
TPRC vs ETRC									
Kulkarni et al. [46]	India	2018	Retrospective Cohort Study	1999–2009	Total: 338 EPRC: 180 TPRC: 158	EPRC: 63 (IQR: 57–73) TPRC: 61 (IQR: 54–79)	NA	EPRC: 72 (IQR: 60–83) TPRC: 70 (IQR: 62–78)	Total: 10 EPRC: 4 TPRC: 6
ET vs T-limb									
Osman et al. [21]	Egypt	2009	RCT	2005–2006	Total: 60 ET: 30 T-limb: 30	ET: 56±8 T-limb: 58±8	ET: 23:77% T-limb: 18:60%	ET: 6±1 T-limb: 7±2	Total: 1 ET: 1 T-limb: 0
Length of ureter									
Richards et al. [59]	USA	2015	Retrospective Case-control Study	2007–2012	Total: 463	68 (IQR: NA)	369:80%	15 (IQR: 7–31)	Total: 25 Ileal conduit: 12 Orthotopic neobladder: 13
Others									
Liu et al. [3]	China	2022	Retrospective Cohort Study	2014–2019	198	67 (IQR: 43–92)	159:80%	NA	Total: 22

Values are presented as mean ± SD, median (IQR), or Category X: XX% as reported in each study.

UIA = ureteral-ileal anastomotic stricture; RARC = robotic-assisted radical cystectomy; LRC = XXX; ORC = open radical cystectomy; IQR = interquartile range; NA = not applicable; RCT = randomized controlled trials; ECUD = extracorporeal urinary diversion; ICUD = intracorporeal urinary diversion; ICG = indocyanine green; SJ = single J; DJ = double J; ST = submucosal tunnel; ET = extramural tunnel; EPRC = extraperitoneal radical cystectomy; TPRC = transperitoneal radical cystectomy.

### 3.2 Risk of bias

The non-randomized studies showed low (one), moderate (34), and high (four) risk of bias (RoB), respectively. Three RCTs were evaluated as having low RoB, and moderate RoB for one study. The case-control studies were marked as a total score of seven for two studies and score of six for one study (Supplementary Tables 4, 5, 6).

### 3.3 RARCs versus ORC

**Fig. 2:**
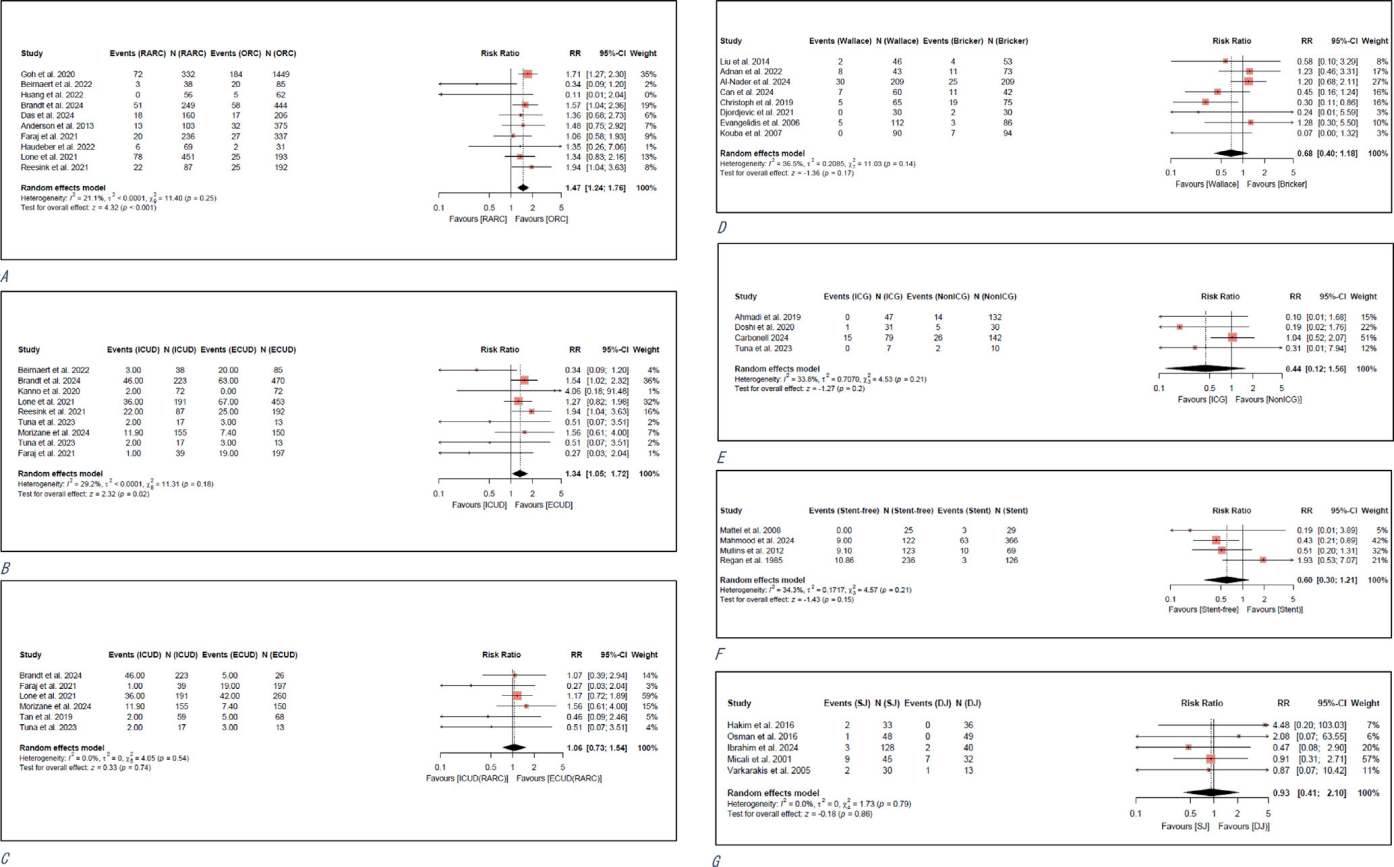
Meta-analyses of different comparisons for the risk of ureteral-ileal anastomotic stricture: (A) RARC versus ORC, (B) ICUD versus ECUD, (C) RARC + ICUD versus RARC + ECUD, (D) Wallace versus Bricker, (E) ICG versus non-ICG, (F) Stent-free versus Stent, (G) SJ stent versus DJ stent. CI = confidence interval; RARC = robotic-assisted radical cystectomy; ORC = open radical cystectomy; ICUD = intracorporeal urinary diversion; ECUD = extracorporeal urinary diversion; ICG = indocyanine green; SJ = single J; DJ = double J.

Among 10 studies (*n* = 4122), the risk of UIAS was compared between RARC and ORC [4], [7], [18], [24], [29], [31], [32], [56], [57], [58]. RARC was significantly associated with a higher risk of UIAS compared with ORC (RR: 1.47; 95% CI: 1.24–1.76; *p* < 0.001). There was no evidence for significant heterogeneity. However, the prospective cohort study conducted by Beirnaert et al. [24] and the RCT by Huang et al. [18] demonstrated differing results in the forest plot, both favoring RARC (RR: 0.34; 95% CI: 0.09–1.20 and RR: 0.11; 95% CI: 0.01–2.04, respectively). Visual inspection of the funnel plot indicated that two of the three smaller studies exhibited a protective effect, which may suggest asymmetry. These observations imply the possibility of publication bias; however, Egger's test yielded a statistically significant result (*p* = 0.013), suggesting the presence of potential small-study effects (Fig. 2A; Supplementary Fig. 3.2A).

### 3.4 ICUD versus ECUD

Among nine studies (*n* = 2581), the risk of UIAS between ICUD and ECUD was compared [7], [24], [28], [31], [32], [39], [42], [50], [57]. ICUD was significantly associated with a higher risk of UIAS compared to ECUD (RR: 1.34; 95% CI: 1.05–1.72; *p* = 0.020). There was no evidence for significant heterogeneity. Visual inspection of funnel plot revealed a symmetric distribution, suggesting a low risk of publication bias. However, in an exploratory analysis of six studies (*n* = 1398) that included only patients undergoing RARC, no significant difference was found between the two groups (RR: 1.06; 95% CI: 0.73–1.54; *p* = 0.5) [7], [28], [31], [57]. There was no evidence found for significant heterogeneity (Fig. 2B and Fig. 2C; Supplementary Fig. 3.2B, 3.2C).

### 3.5 Wallace versus Bricker

Among eight studies (*n* = 1678), the risk of UIAS between Wallace and Bricker methods was compared [22], [26], [27], [33], [34], [38], [44], [51]. No significant difference was observed between the two methods (RR: 0.68; 95% CI: 0.40–1.18; *p* = 0.17). There was no evidence found for significant heterogeneity (Fig. 2D; Supplementary Fig. 3.2D).

### 3.6 ICG versus non-ICG

Among five studies (*n* = 572), the impact of ICG on the risk of UIAS was evaluated [37], [40], [50], [53], [55]. No significant difference was observed between the two (RR: 0.44; 95% CI: 0.12–1.56; *p* = 0.2). There was no evidence found for significant heterogeneity. Shen et al. [40] 2019 (*n* = 94) found that stricture occurred in seven out of 93 ureters (7.5%) without using ICG compared to zero out of 93 in ICG group. This study was not included in analysis because the assessment was reported per ureter instead of per patient (Fig. 2E; Supplementary Fig. 3.2G) [40].

### 3.7 Stent-free versus stent

Among five studies (*n* = 1096) the risk of UIAS in stent-free patient group was evaluated [19], [35], [43], [48]. No significant difference was found between the stent-free and stented group (RR: 0.60; 95% CI: 0.30–1.21; *p* = 0.15). There was no evidence found for significant heterogeneity (Fig. 2F; Supplementary Material).

### 3.8 SJ stent versus DJ stent

Among five studies (*n* = 450) the risk of UIAS between the SJ stent and DJ stent group was compared [9], [20], [23], [30], [52]. There was no significant difference observed between the two groups (RR: 0.93; 95% CI: 0.41–2.10; *p* = 0.9). There was no evidence found for significant heterogeneity here as well (Fig. 2G; Supplementary Fig. 3.2F).

### 3.9 Reflexing versus non-reflexing ureter

Four studies (*n* = 386) reported on the differences in UIAS between patients treated with refluxing and non-refluxing ureters (Table 1). Pantuck et al. [49] examined 58 patients and found that the incidence of stricture was 13% (8/60) of ureters with non-refluxing implantation, compared to only 1.8% (1/56) of refluxing ureters. Similar results were observed in a previous study by Roth et al. [54], which reported 6.5% (3/46) strictures in refluxing ureters, and 29 strictures in 74 patients with non-refluxing ureters. De Carli et al. [47] reported stricture incidence of 6/50 and 6/52 in non-refluxing and refluxing group respectively. Conversely, Hautman et al. [41] reported higher incidence of strictures in refluxing ureters (30% [11/37]), compared to non-refluxing (15% [10/69]). A meta-analysis was not performed due to inconsistencies in outcome assessment, as some studies evaluated UIAS based on the number of ureters, while others assessed it per patient.

### 3.10 Running versus Interrupted sutures

Large et al. (2013) conducted a retrospective cohort study from 2007 to 2010, comprising 158 patients. The study compared two suturing techniques: Interrupted (with 149 patients) and Running (with 109 patients). In the Interrupted group, there were 25 cases of strictures, while the Running group had 27 cases (Table 1) [45]. Running anastomosis was associated with UIAS stricture (Hazards ratio: 1.92; 95% CI: 1.00–3.70; *p* = 0.050).

## 4 Discussion:

In this systematic review and meta-analysis, we aimed to evaluate the impact of various surgical techniques on the incidence of UIAS in UD following RC. Our analysis yielded several key findings. First, RARC was significantly associated with a higher risk of UIAS compared to ORC. Second, ICUD was associated with a higher incidence of UIAS compared to ECUD, except for an analysis that was restricted to patients undergoing RARC, in which no significant difference was found between ICUD and ECUD. Third, among several modifications investigated, none appeared to be significantly associated with the risk of stricture.

In addition to the increased risk of UIAS identified in our study, we found that RARC was associated with a 47% increase in the risk of UIAS compared with ORC. A systematic review and meta-analysis conducted by Novara et al. [60] found that the operative time was significantly longer in the RARC group compared to ORC (mean difference [MD]: 84; 95% CI: 57–110; *p* < 0.01), but RARC resulted in reduced blood loss (MD: −522; 95% CI: −644 to −400; *p* < 0.01) and a lower transfusion rate (OR: 0.16; 95% CI: 0.10–0.27; *p* < 0.01). Moreover, complications at 90-d were significantly lower in the RARC group compared to the ORC group for both with any grade complications (OR: 0.44; 95% CI: 0.31–0.61; *p* < 0.001) and grade 3 complications (OR: 0.55; 95% CI: 0.35–0.98; *p* = 0.04). However, the prospective cohort study conducted by Beirnaert et al. [24] and the RCT by Huang et al. [18] demonstrated differing results in the forest plot, both favoring RARC (RR: 0.34; 95% CI: 0.09–1.20 and RR: 0.11; 95% CI: 0.01–2.04, respectively), These methodological differences may contribute to variability in reported outcomes. RCTs and prospective studies generally provide stronger control over bias than retrospective analyses, which may influence the observed effect sizes. The predominance of retrospective data underscores the need for cautious interpretation, as residual confounding and selection bias cannot be excluded. Future research should prioritize prospective, multicenter RCTs to validate these findings and enhance the robustness of evidence comparing RARC and ORC. Considering these findings, RARC offers potential benefits due to smaller incisions, high-definition vision, and favorable surgeon ergonomics [60]. Despite the potential risk for UIAS, the demand for RARC will likely continue to increase in the future. However, our findings raise awareness regarding the risk of stricture, which appears to be more prevalent using this approach. Therefore, efforts to reduce the risk of UIAS by refining other procedural aspects should be encouraged. One of the possible explanations for the increased risk of UIAS associated with robotic-assisted surgery is limited tactile feedback that robotic systems offer. This makes it more challenging to detect subtle tissue tension or ischemia during surgery. Additionally, ureteral thermal injury can occur from excessive cautery or heat during the robotic dissection of the ureter. Moreover, the robotic technique’s precision, while beneficial in many aspects, could also lead to increased tension at the anastomosis site due to the inability to precisely adjust for anatomical variations. Furthermore, factors such as intraoperative fluid dynamics and learning curve effects might play a role in increasing the incidence of UIAS.

ICUD was associated with 34% higher risk of UIAS compared with ECUD, but in an analysis restricted to patients undergoing RARC, no significant difference in UIAS incidence was found among both. With the development of robotic surgery, there has been a shift from ECUD to ICUD as a less invasive approach. Feng et al compared perioperative outcomes between RARC + ECUD (*n* = 170) and RARC + ICUD (*n* = 1271). Although they assumed that ICUD might reduce the risk of UIAS by 64% (RR: 0.36; 95% CI: 0.14–0.91; *p* = 0.03), no significant differences were observed in operative time (MD: 0.17; 95% CI: −0.38 to 0.73; *p* = 0.6), blood transfusion (RR: 0.52; 95% CI: 0.23–1.13; *p* = 0.10), and 30-d readmission (RR: 0.80; 95% CI: 0.60–1.06; *p* = 0.12). The discrepancy between the overall comparison of ICUD versus ECUD and the subgroup analysis restricted to RARC may be explained by the potential benefits of ORC rather than the differences between ICUD and ECUD [61]. Therefore, in patients undergoing RARC, ICUD appears to be a viable approach with no clear evidence for an increased risk of UIAS.

We also found no significant difference in the risk of UIAS between UD with or without ICG. Use of ICG showed a trend reducing the risk of UIAS without reaching statistical significance, whereas individual analysis did not show a significant reduction. Although the precise etiology of UIAS remains unclear, it is often linked to inadequate blood supply to the anastomotic site, urine leakage, and fibrosis formation [62], [63], [64]. ICG provides real-time visualization of blood vessels and tissue perfusion, enabling the surgeons to accurately assess blood flow at the anastomosis site and ensures sufficient perfusion [10]. Furthermore, a meta-analysis by Fan et al reported that the use of ICG was also associated with a significant reduction in the risk of overall severe complications [65]. Considering these findings, ICG could be considered a valuable tool regardless of its non-significant effect on UIAS.

No significant difference was found in the risk of UIAS between Wallace and Bricker techniques. Although, the pooled RR for Wallace versus Bricker anastomosis did not reach statistical significance (RR: 0.68; 95% CI: 0.40–1.18; *p* = 0.17), forest‐plot inspection shows that six of eight studies report point estimates favoring Wallace, with several confidence intervals close to null threshold. This suggests a potential clinical benefit of the Wallace technique that warrants confirmation in larger prospective trials. However, these findings may be influenced by confounding factors related to other surgical techniques, such as RARC versus ORC, ICUD versus ECUD, and running versus interrupted sutures, and their careful interpretation is necessary. In the absence of robust evidence, each technique has its advantages and disadvantages, and the optimal approach remains controversial. The choice of technique largely depends on the surgeon’s experience and preference as well.

No significant differences were found in UIAS risk between stent-free and stent approaches, as well as DJ versus SJ stents. Ureteral stents are widely utilized in UD to prevent obstruction of ureters, maintain anastomotic alignment, and minimize the risk of urine leakage. However, there have been some concerns that stent placement can lead to bacterial colonization and increase the risk of pyelonephritis due to the reflux of contaminated urine from the bowel segments. In this context, some surgeons prefer to perform a stent-free UD to avoid infection-related complications and subsequent UIAS formation. Our analysis suggests that stent-free UD does not provide a protective effect against UIAS. Moreover, considering its other advantages, such as preventing leakage and its associated complications, there appears to be no justification for recommending a stent-free approach at present [48], [66], [67]. Although commonly used DJ stents were previously used, clinical practice has shifted toward SJ stents. No significant differences were found in complications, including UIAS. Considering the potential benefit of eliminating the necessity for a follow-up surgery to remove the stent, SJ stents may still be preferable [9].

The current study also has several limitations. First, 38 out of 46 included studies are retrospective. Given the retrospective nature of the included studies, it is plausible that UIAS rates may be underreported due to inconsistent follow-up protocols or documentation practices. This potential bias could affect the accuracy of pooled estimates, and may not be uniformly distributed across centers, surgical techniques, or patient populations—highlighting the need for cautious interpretation and further prospective validation. Second, selection bias in the choice of surgical technique may have influenced the results, particularly if certain approaches were preferentially applied to higher-risk patients based on clinical judgment or institutional protocols. Most included studies did not employ adjustment methods such as propensity score matching or inverse probability treatment weighting, limiting our ability to account for confounding variables and assessing whether this bias was evenly distributed across centers, surgical approaches, or patient subgroups. RARC involves multiple technical steps, and the selection of specific techniques may have differed among institutions and surgeons. This variation could have contributed to the selection bias and affected the observed outcomes. Third, RC and UD are complex procedures involving multiple steps, each of which can be performed in various ways. Fourth, evidence of publication bias was observed in the RARC versus ORC comparison. This may reflect selective reporting of favorable perioperative outcomes, under‑publication of studies with negative or null findings, and the predominance of single‑center experiences with relatively small sample sizes. Additionally, industry sponsorship and the novelty of robotic surgery may have contributed to preferential dissemination of positive results. These factors should be considered when interpreting the pooled estimates, as they may exaggerate the apparent advantages of RARC. Consequently, confounding factors may have influenced our findings. For instance, in the comparison between ICUD and ECUD, we observed a significant higher risk of UIAS with ICUD in the overall population. However, when restricted to patients undergoing RARC, this difference was no longer statistically significant. Additionally, factors such as the surgeon's experience and technical proficiency may have further impacted outcomes. Moreover, it is important to acknowledge the diversity present across the included studies. The surgical techniques employed varied, including RARC versus ORC and ICUD versus ECUD. Additionally, patient characteristics such as age, comorbidities, and cancer staging also differed among these studies. This variability could influence the results and should be considered while interpreting our findings. Future research would benefit from using more consistent surgical methods and patient populations to help clarify these effects. Conflicting directional findings: A minority of comparisons yielded RRs crossing the null value in the opposite direction, reflecting inconsistency in the evidence base and limiting definitive conclusions. Finally, although studies investigated other key surgical techniques such as running versus interrupted sutures, retrosigmoid versus anterosigmoid transposition, and submucosal tunnel versus extramucosal tunnel, the number of available studies remains limited, making it difficult to draw definitive conclusions. Future research should focus more on prospective, RCTs to validate our findings and explore the impact of surgical techniques on UIAS.

## 5 Conclusion:

The current study found that RARC is associated with a significantly higher risk of UIAS compared with ORC. While the shift from ECUD to ICUD continues alongside the growing adoption of RARC, driven by its advantages in other areas. However, we did not observe a significant difference in UIAS incidence among patients undergoing RARC. It is important to acknowledge the potential influence of confounding factors on these findings. Despite extensive exploration of various approaches to mitigate the incidence of UIAS, none of the approaches used have demonstrated clear superiority in reducing its occurrence. These approaches include comparisons between Wallace and Bricker techniques, the use of ICG, double versus single J stents—or even no stent at all—and refluxing versus non-refluxing ureters. Additionally, the current body of research on surgical techniques directly related to anastomosis, such as suturing methods and tunneling approaches, remains limited. To advance the field and enhance patient outcomes, further well-designed, prospective studies exploring diverse surgical techniques are essential.

  ***Author contributions***: Shahrokh F. Shariat had full access to all the data in the study and takes responsibility for the integrity of the data and the accuracy of the data analysis.

  *Study concept and design*: Ahmed Riyadh Alfarhan, Akihiro Matsukawa, Shahrokh F. Shariat.

*Acquisition of data*: Ahmed Riyadh Alfarhan, Akihiro Matsukawa.

*Analysis and interpretation of data*: Ahmed Riyadh Alfarhan, Akihiro Matsukawa.

*Drafting of the manuscript*: Ahmed Riyadh Alfarhan, Akihiro Matsukawa, Angelo Cormio, Marcin Miszczyk, Abdulrahman A. Alhazeem, Abdulrahman S. Alqathtani, Alessandro Dematteis, Ichiro Tuboi, Navid Roessler, Takafumi Yanagisawa, Jun Miki, Piotr Chłosta, Pierre I. Karakiewicz, Takahiro Kimura.

*Critical revision of the manuscript for important intellectual content*: Ahmed Riyadh Alfarhan, Akihiro Matsukawa, Angelo Cormio, Marcin Miszczyk, Abdulrahman A. Alhazeem, Abdulrahman S. Alqathtani, Alessandro Dematteis, Ichiro Tuboi, Navid Roessler, Takafumi Yanagisawa, Jun Miki, Piotr Chłosta, Pierre I. Karakiewicz, Takahiro Kimura, Shahrokh F. Shariat.

*Statistical analysis*: Ahmed Riyadh Alfarhan, Akihiro Matsukawa.

*Obtaining funding*: None.

*Administrative, technical, or material support*: None.

*Supervision*: Shahrokh F. Shariat.

*Other* (specify): None.

  ***Financial disclosures:*** Shahrokh F. Shariat certifies that all conflicts of interest, including specific financial interests and relationships and affiliations relevant to the subject matter or materials discussed in the manuscript, are as follows: Honoraria: Astellas, AstraZeneca, BMS, Ferring, Ipsen, Janssen, MSD, Olympus, Pfizer, Roche, Takeda Consulting or Advisory Role: Astellas, AstraZeneca, BMS, Ferring, Ipsen, Janssen, MSD, Olympus, Pfizer, Pierre Fabre, Roche, Takeda Speakers Bureau: Astellas, AstraZeneca, Bayer, BMS, Ferring, Ipsen, Janssen, MSD, Olympus, Pfizer, Richard Wolf, Roche, Takeda Takahiro Kimura is a paid consultant/advisor for Astellas, Bayer, Janssen, and Sanofi. Marcin Miszczyk was supported by the European Urological Scholarship Programme (EUSP) / EAU Scholarship. The other authors declare no conflicts of interest.

  ***Funding/Support and role of the sponsor*:** No external funding was provided.

  ***Acknowledgment:*** None.

## Appendix A Supplementary material

The following are the Supplementary data to this article includes detailed methodological checklists (PRISMA 2020, PRISMA for Abstracts, PICOS), risk of bias assessments (ROBINS‑I, RoB 2, NOS), subgroup forest plots, sensitivity analyses, funnel plots, Egger’s test, and full search strategies and AMSTAR‑2 checklist used in the systematic review and meta-analysis:
